# Error in Figure 1

**DOI:** 10.1001/jamanetworkopen.2025.43834

**Published:** 2025-10-22

In the Original Investigation titled “Cardiopulmonary Point-of-Care Ultrasonography for Hospitalist Management of Undifferentiated Dyspnea,”^1^ published September 5, 2025, there was an error in Figure 1. The number excluded from the standard-of-care approach was listed as 71 participants instead of 7 participants. This article has been corrected.^1^

## References

[1] Maganti K, Chen C, Jamthikar AD, Parikh P, Yanamala N, Sengupta PP. Cardiopulmonary point-of-care ultrasonography for hospitalist management of undifferentiated dyspnea. JAMA Netw Open. 2025;8(9):e2530677. doi:10.1001/jamanetworkopen.2025.30677 40911308 PMC12413653

